# Clinical utility of a 1.5 T magnetic resonance imaging-guided linear accelerator during conventionally fractionated and hypofractionated prostate cancer radiotherapy

**DOI:** 10.3389/fonc.2022.909402

**Published:** 2022-08-16

**Authors:** Gorkem Turkkan, Nazli Bilici, Huseyin Sertel, Yavuz Keskus, Sercan Alkaya, Busra Tavli, Muge Ozkirim, Merdan Fayda

**Affiliations:** ^1^ Department of Radiation Oncology, Istinye University Faculty of Medicine, Istanbul, Turkey; ^2^ Department of Radiation Oncology, Liv Hospital Ulus, Istanbul, Turkey

**Keywords:** adaptive radiotherapy, fractionated radiotherapy, MRI-guided radiotherapy, MRI-LINAC, prostate cancer

## Abstract

**Purpose:**

To report our initial experience with 1.5 T magnetic resonance imaging (MRI) linear accelerator (LINAC) in prostate cancer radiotherapy in terms of its use in a radiation oncology clinic.

**Methods:**

The medical records of 14 prostate cancer patients treated with MRI-guided radiotherapy were retrospectively evaluated. The fraction time, adapt-to-position (ATP):adapt-to-shape (ATS) usage rate, machine-associated treatment interruption rate, median gamma pass rate, the percentage of planning target volume receiving at least 95% of the prescription dose coverage value of each ATS fraction, the effect of the learning curve on the fraction time and radiation-related acute gastrointestinal and genitourinary toxicities were evaluated.

**Results:**

Fourteen patients have completed their treatment receiving a total of 375 fractions. Six patients (42%) were treated with the moderately hypofractionated regimen, five patients (36%) with conventionally fractionated, and three patients (22%) with the ultra-hypofractionated radiotherapy regimens. The ATP : ATS usage ratio was 3:372. The median fraction time was 46 min (range, 24-81 min). For the 3%/3 mm criterion, median gamma pass rate was 99.4% (range, 94.6–100%). Machine-related treatment interruptions were observed in 11 (2.9%) of 375 fractions, but this interruption rate decreased from 4.1% to 0.8%, after an upgrade. Three patients (22%) had gastrointestinal and five patients (36%) had genitourinary toxicity. No ≥grade 3 toxicity was observed.

**Conclusion:**

1.5 T MRI-LINAC device could be used as a conventional LINAC device, when the conditions of the radiotherapy center are appropriate. MRI-guided prostate radiotherapy is safe and feasible, and high-quality studies with a larger number of patients and long-term results are needed to better evaluate this new technology.

## Introduction

Prostate cancer is responsible for 26% of new cancer diagnoses and 11% of cancer-related deaths among men (1). Radiotherapy plays an important role, and magnetic resonance imaging-guided linear accelerator (MRI-LINAC) devices are increasingly being used in the treatment of prostate cancer.

Unity MRI-LINAC (Elekta, AB, Stockholm, Sweden) integrates a high-field 1.5 Tesla MRI with a LINAC device and allows online adaptive radiation treatments with the guidance of high-quality MR images. This technology can provide more accurate radiotherapy treatments than ever before, especially in areas where soft tissues are dominant, because of the high-quality image guidance of MRI and online adaptive workflow.

Theoretically, better visualization and online tracking of the prostate and organs at risk during radiation treatment and adaptive planning options may pave the way for achieving higher tumor control with less toxicity, as a result of increasing radiation doses and reducing margins for prostate radiotherapy. These advantages can also enable safer application of moderate- and even ultra-hypofractionation (2–4). In addition, taking daily MR images during each radiotherapy fraction supports the possibility of achieving more optimal treatment, and obtaining diffusion-weighted images may enable the assessment of the biological response of the patient (5).

In this study, we aimed to report our initial experience with 1.5 T MRI-LINAC in prostate cancer radiotherapy in terms of its use in clinical practice, including the treatment session duration, machine-associated treatment interruption rate, dose delivery success, and treatment-related early side effects.

## Materials and methods

This retrospective study included patients with prostate cancer treated with curative-intent radiotherapy using MRI-LINAC at our institution between August 2021 and February 2022. All the patients had histologically confirmed prostate adenocarcinoma and a Karnofsky performance score ≥80 (Eastern Cooperative Oncology Group [ECOG] performance status ≤1). None of the patients had a history of radiotherapy to the pelvic region or MRI contraindications, such as claustrophobia, cardiac pacemakers, or other metallic surgical implants. The study was approved by the institutional ethics committee and was conducted in accordance with the Declaration of Helsinki and good clinical practice. Informed consent was obtained from all the patients.

All the patients were informed of the necessary procedures prior to simulation. The patients were encouraged to drink two glasses of water after full urination (as a routine, 300 cc of water 60 min before each session) and to empty their rectum (defecation, laxatives/enemas when needed) before simulation imaging (planning computed tomography [CT]/MRI) and each radiotherapy fraction. When needed, an MRI-compatible plastic penile clamp was used in patients who had urinary incontinence problems. For each patient, a CT scan with a slice thickness of 2.5 mm was performed to calculate the radiation doses in treatment planning. For patients with non-optimal bladder volume (i.e.<300 cc) or without acceptable rectal conditions, a new CT scan was performed after obtaining reasonable conditions. Additionally, high-quality MRI scans with 3D T2-weighted and diffusion-weighted images were obtained by Elekta Unity.

Radiotherapy schemes varied from conventionally fractionated (CF: 66–78 Gy/36–42 fractions, 5 fractions per week) to moderately hypofractionated (MHF: 70 Gy/28 fractions, 5 fractions per week) and ultra-hypofractionated/stereotactic body radiation therapy (UHF/SBRT: 36.25 Gy/5 fractions, 2 fractions per week, on Mondays and Thursdays or on Tuesdays and Fridays) regimens. Node-positive patients and patients requiring salvage radiotherapy with or without measurable relapsed tumors were treated using CF regimens. The clinical target volume (CTV) included prostate+seminal vesicles/prostate+seminal vesicle bed; clinically/radiologically gross tumor area (if present); and presacral, internal iliac, external iliac, and obturator lymph node areas. The planning target volume (PTV) was created by adding 10 mm-margins in each direction to the lymph node areas and 5 mm-margins in each direction to the prostate and seminal vesicles bed/region and clinically/radiologically gross tumor areas, except for 3 mm posteriorly in phase I (up to 45 Gy), and 5 mm-margins in each direction to the prostate/prostate bed, except for 3 mm posteriorly in phase II. Treatment plans were generated using the Monaco (Elekta AB, Stockholm, Sweden, v5.51.10) treatment planning system in accordance with previous recommendations (6).

The remaining patients were treated using hypofractionated regimens. The MHF or UHF/SBRT regimens were chosen based on patient and disease characteristics. The CTV for hypofractionated regimens included the prostate alone, prostate plus proximal 1-cm seminal vesicles, and prostate plus seminal vesicles for patients with low-risk, intermediate-risk, and high-risk/oligometastatic disease, respectively. The PTV was defined as the CTV plus 5 mm-margins in each direction, except 3 mm posteriorly. The treatment plans were generated using the Monaco (Elekta AB, Stockholm, Sweden, V5.51.10) treatment planning system in accordance with previous recommendations (7).

Elekta Unity allows two different options for daily plan adaptation: adapt-to-shape (ATS) and adapt-to-position (ATP). The ATS option uses deformable image registration to project contours from the reference plan to the corresponding MR session, permits re-adjustment of the contours if necessary, and thus enables the creation of a new plan that matches the daily anatomy. On the other hand, the ATP option allows for the repositioning of the isocenter location in the reference plan, aligning with the instant positions of the target volumes and organs at risk (OARs). Contour rearrangement is neither possible nor necessary in ATP. In this study, almost all treatments were completed using ATS. Delineations were edited, and the relevant air contours close to the PTVs were added to consider the electron returning effect, if necessary. Immediately after, a re-optimization was performed, and a verification MRI scan was acquired to ensure that the target volumes and OARs were still in an appropriate position/status. If everything was appropriate, the daily radiation dose was administered using motion monitoring, which allowed instant checking of the positions of the target volume(s) and OARs in the sagittal, axial, and coronal planes. In the presence of an inappropriate condition, the patient was repositioned for treatment after the necessary preparations were made.

The technical data regarding the daily practice of MRI-guided prostate cancer radiotherapy were reviewed. The fraction time, ATP : ATS usage rate, machine-associated treatment interruption rate, and median gamma pass rate were determined. The percentage of PTV receiving at least 95% of the prescription dose (PTV95%) coverage value of each ATS fraction was compared with its equivalent in the reference plan. The fraction time was defined as the time from entering the treatment room to leaving the treatment room. Additionally, the elapsed time between the first and last fractions in the study was divided into half to assess the effect of the learning curve on the fraction time. Notably, the approximate time required to achieve optimal bladder filling was determined for each patient based on the images obtained in the first treatment fractions.

In our daily practice, the number and causes of treatment interruptions are regularly noted during each fraction. Machine-associated interruptions comprise hardware- and software-associated problems. Patient-related interruptions were noted separately. Each of these interruptions completely stopped the treatment, resulting in the restarting of the workflow of the relevant fraction. No completion plan had been planned. When calculating the fraction time for the relevant fraction, only the time that elapsed during the new workflow was considered.

For each treatment, the intensity-modulated radiotherapy quality assurance measurements were performed using an ArcCheck MR phantom (Sun Nuclear Corporation, Melbourne, FL, USA). The gamma analysis results were measured in the ArcCHECK R phantom diodes and analyzed with regard to the agreement between the reference doses that were calculated in the treatment planning system. Gamma evaluations were performed considering the 3%/3 mm criterion. Assessments were performed in the absolute mode with normalization to the maximum dose, using a 5% threshold to limit the analysis to the clinically relevant area.

In addition, during (once a week or on the patient’s request) and at the end of the treatment, radiation-related acute gastrointestinal and genitourinary toxicities were assessed by the clinician according to the Common Terminology Criteria for Adverse Events (CTCAE) scale, v5.0.

All statistical analyses were performed using the SPSS software version 22.0; (SPSS Inc., Chicago, IL, USA). A descriptive analysis was performed for the demographic and clinical characteristics in the study, with the mean, median, and range.

## Results

A total of 14 prostate cancer patients completed their treatment, receiving a total of 375 fractions within 6 months. The median age was 71 years, and most patients (71%) had an ECOG performance status of 0. Of the 14 patients, six patients (42%) had localized disease, three patients (22%) had recurrent disease after radical prostatectomy (two patients with biochemical recurrence and one patient with macroscopic local recurrence), three patients (22%) had oligometastatic disease, and two patients (14%) had node-positive disease. Six patients (42%) were treated with the MHF regimen, five patients (36%) with CF, and three patients (22%) with the UHF/SBRT regimen. All patients treated with the UHF/SBRT regimen had oligometastatic disease. The patient and treatment characteristics are summarized in **Table 1**.

**Table 1:** Patient and treatment characteristics.

Characteristic	n (%)
Age (median, range)	71 (60-84)
The ECOG performance status 0 1	10 (71%) 4 (29%)
Disease definition Localized disease Node-positive disease Recurrent disease after radical prostatectomy Oligometastatic disease	6 (42%) 2 (14%) 3 (22%) 3 (22%)
RT fractionation classification, radiation field CF, local RT plus PLNI MHF, local RT UHF/SBRT, local RT	5 (36%) 6 (42%) 3 (22%)
Used RT scheme, radiation field 78 Gy/42 fractions, local RT plus PLNI 66-72 Gy/36 fractions, local RT plus PLNI 70 Gy/28 fractions, local RT 36.25 Gy/5 fractions, local RT	2 (14%) 3 (22%) 6 (42%) 3 (22%)
Androgen deprivation therapy Yes No	10 (71%) 4 (29%)

CF, conventionally fractionated; ECOG, Eastern Cooperative Oncology Group; Gy, gray; MHF, moderately hypofractionated; PLNI, pelvic lymph node irradiation; RT, radiotherapy; UHF/SBRT, ultra-hypofractionated/stereotactic body radiation therapy.

**Figure 1:**
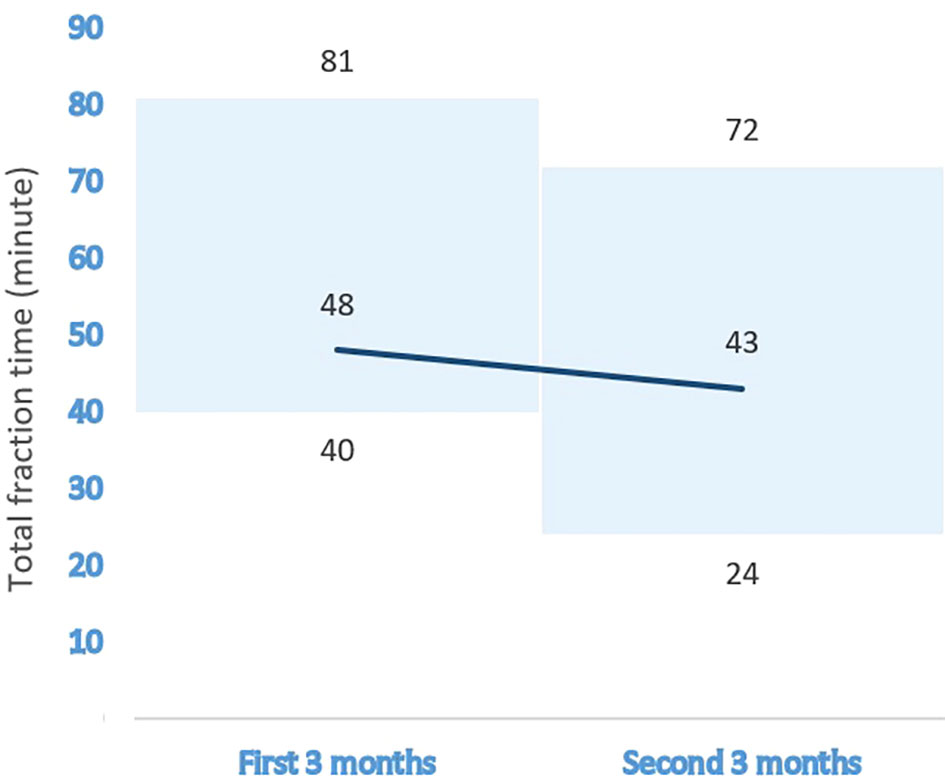
The effect of the learning curve on the median treatment time for MRI-guided prostate cancer radiotherapy.

The median time required for the patients’ bladders to reach optimal fullness was 80 min (range, 60–240 min). **Table 2** summarizes the demographics, disease characteristics, and treatment procedures of each patient. For daily plan adaptation, the ATP : ATS ratio was 3:372. With the use of the “optimize shapes” adaptive calculation method, an excellent agreement was achieved with a mean PTV 95% coverage value of 99.2% (range, 97.6–102.0%), when the PTV 95% values in 372 ATS fractions were compared to those in the reference treatment plans of the relevant patients. The median fraction time was 46 min (range, 24–81 min) for the entire group. When classified into three subgroups, these values were 49, 42, and 54 min for CF, MHF, and UHF/SBRT regimens, respectively. Regarding the effect of the learning curve on the fraction time, 180 fractions were applied in the first half and 195 fractions in the second half of the total duration of the study. The median fraction times for the first and second halves of the total study duration were 48 min (range, 40–81 min) and 43 min (24–72 min), respectively. **Figure 1** shows the effect of the learning curve on the median treatment time for MRI-guided prostate cancer radiotherapy.

**Table 2:** Detailed summary of each patient’s demographics, disease characteristics, and treatment procedures.

Patient	Age	Comorbidity	Oncological treatment before RT	RT scheme	RT field	Optimal bladder filling time (min)
**1**	84	Hypertension, DVT	RP plus LND	72 Gy/36 fr	Local RT plus PLNI	115
**2**	76	Hypertiroidism, left radical nephrectomy, bilateral hip prosthesis	None	78 Gy/42 fr	Local RT plus PLNI	100
**3**	64	None	RP plus LND	66 Gy/36 fr	Local RT plus PLNI	80
**4**	69	Chronic obstructive pulmonary disease	Robotic RP plus LND	66 Gy/36 fr	Local RT plus PLNI	60
**5**	71	Coronary artery disease (By-pass)	None	78 Gy/42 fr	Local RT plus PLNI	60
**6**	66	Parkinson disease	None	70 Gy/28 fr	Local RT	150
**7**	77	Hypertension	None	70 Gy/28 fr	Local RT	60
**8**	80	Polisitemia vera, hypertension	None	70 Gy/28 fr	Local RT	80
**9**	62	Parkinson disease	None	70 Gy/28 fr	Local RT	240
**10**	70	Hypertension, diabetes mellitus, obstructive sleep apnea syndrome, coronary artery disease (By-pass), brain aneurysm,	None	70 Gy/28 fr	Local RT	60
**11**	83	Hypertension	None	70 Gy/28 fr	Local RT	80
**12**	60	Hypertension, arrhythmia	ADT, ChT, zoledronate	36.25 Gy/5 fr	Local RT	90
**13**	66	None	ADT	36.25 Gy/5 fr	Local RT	60
**14**	72	Hypertension, diabetes mellitus, coronary artery disease (By-pass)	ADT, ChT	36.25 Gy/5 fr	Local RT	60

ADT, androgen deprivation therapy; ChT, chemotherapy; DVT, deep vein thrombosis; Fr, fraction; Gy, gray; LND, lymph node dissection; PLNI, pelvic lymph node irradiation; RP, radical prostatectomy; RT, radiotherapy.

**Figure 2:**
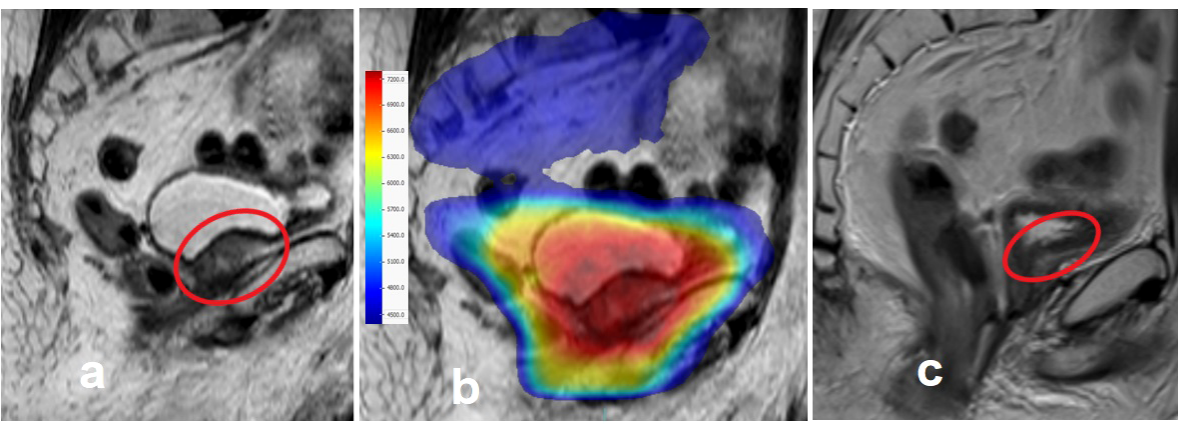
An exemplary case treated with MRI-guided prostate cancer radiotherapy. **(A)** Macroscopic prostate bed relapse near the intestines 15 years after radical prostatectomy. **(B)** The sum of dose distributions to the pelvic lymph nodes, prostate bed, and relapse area. **(C)** Total macroscopic regression of the tumor, 3 months after radiotherapy.

Machine-related treatment interruptions were observed in 11 (2.9%) of 375 fractions. A median gamma pass rate of 99.4% (range, 94.6–100%) for the 3%/3 mm criterion was found, and the compatibility between the calculated and measured dose distributions was perfect. The dose distribution and treatment response images of a patient with macroscopic relapse in the prostate bed treated with the CF regimen to the pelvic lymph nodes plus the prostate bed area are shown in **Figure 2**.

Approximately 4 months after starting MRI-guided radiotherapy treatments, an upgrade providing software and hardware changes (Unity 2.0.2.0 maintenance release) was performed to improve the performance and reliability of the entire system. Based on the date of this upgrade, both treatment fractions and machine-related treatment interruptions were divided into two subgroups: before and after the upgrade. As a result, it was observed that the machine-associated treatment interruption rate decreased from 4.1% (before upgrade) to 0.8% (after upgrade). Technical data including detailed information on machine-associated treatment interruption during prostate radiotherapy with 1.5 T MRI-LINAC is shown in **Table 3**.

**Table 3:** Technical data for prostate radiotherapy with 1.5 T MRI LINAC.

Parameter	Value
ATP : ATS usage ratio	3:372
Total fraction count CF (local RT plus PLNI) MHF (local RT) UHF/SBRT (local RT)	192 (51%) 168 (45%) 15 (4%)
Fraction time in minutes (median, range) CF (local RT plus PLNI) MHF (local RT) UHF/SBRT (local RT)	49 (30-77) 42 (24-81) 54 (40-72)
Monitor unit value per fraction (mean) CF (local RT plus PLNI) MHF (local RT) UHF/SBRT (local RT)	923.2 854.4 2581.2
Gamma pass rate (%, median, range)	99.4 (94.6-100)
Machine-associated treatment interruption rate Before upgrade: 10 interruptions in 242 fractions After upgrade: 1 interruption in 133 fractions	10 (4.1%) 1 (0.8%)
Machine-associated treatment interruption reasons Mosaiq failure (data transfer fault) Monaco failure (data transfer fault) MRI display connection failure Energy mismatch failure	4 3 3 1

ATP, adapt-to-position; ATS, adapt-to-shape; CF, conventionally fractionated; LINAC, linear accelerator; MHF, moderately hypofractionated; MRI, magnetic resonance imaging; PLNI, pelvic lymph node irradiation; RT, radiotherapy; UHF/SBRT, ultra-hypofractionated/stereotactic body radiation therapy.

Nine patients (64%) underwent complete treatment without any acute toxicity. No grade 3 toxicity was observed. In terms of the gastrointestinal toxicity, one patient (7%) had grade II toxicity and two patients (14%) had grade I toxicity. On the other hand, five patients (36%) had grade I genitourinary toxicity. The treatment-related acute toxicity rates scored by clinicians according to the CTCAE scale (v5.0) are shown in **Table 4**.

**Table 4:** Acute gastrointestinal and genitourinary toxicity rates scored by clinicians according to the CTCAE scale v5.0.

Toxicity Type	Toxicity Grade			
	G0 (n,%)	G1 (n,%)	G2 (n,%)	G3-4 (n,%)
Genitourinary (dysuria, frequent urination, urgency) Local RT Local RT plus PLNI	9 (64%) 7 (50%) 2 (14%)	5 (36%) 2 (14%) 3 (22%)	0 (0%) 0 (0%) 0 (0%)	0 (0%) 0 (0%) 0 (0%)
Gastrointestinal (diarrhea, rectal bleeding, rectal pain) Local RT Local RT plus PLNI	11 (79%) 8 (57%) 3 (22%)	2 (14%) 1 (7%) 1 (7%)	1 (7%) 0 (0%) 1 (7%)	0 (0%) 0 (0%) 0 (0%)

CTCAE, common terminology criteria for adverse events; G, grade; PLNI, pelvic lymph node irradiation; RT, radiotherapy.

## Discussion

To the best of our knowledge, the present study is the first to report on three different dose-fractionation approaches (CF, MHF, and UHF/SBRT) with 1.5 T MRI-LINAC in prostate cancer radiotherapy.

In our daily practice, the ATS option was preferred in nearly all MRI-guided prostate cancer radiotherapy fractions (99.2%), to consider the daily anatomical changes more accurately and benefit from advanced imaging technology at the maximum level (8). This strategy allows replanning at each fraction and paves the way for optimizing doses not only to the target volumes, but also to the OARs. It has also been recently stated that adaptive therapies should be used in especially hypofractionated prostate cancer radiotherapy in order to provide safe and effective treatments, because of the intrafraction motion (-6 mm anterior-posterior translation and -9 deg left-right rotation) (9). Thus, it may be advantageous to patients in terms of better local control and treatment-related side effects. However, choosing the ATP option was possible in only 3 (0.8%) of the 375 fractions.

The median ATS fraction time with a 1.5 T MRI-LINAC reported in the literature varied between 42 and 56 min (10–12). Our median ATS fraction time of 46 min was also within this range. However, it should be noted that the fraction counts in these studies ranged from 125 to 313, and some of these studies included treatments other than curative prostate cancer irradiation. In addition, the current study provides detailed information on the ATS fraction time for CF, MHF, and UHF/SBRT regimens for MRI-guided prostate cancer radiotherapy. This study makes significant contributions to the existing literature. We also observed that the median fraction time decreased from 48 to 43 min based on our learning curve. Therefore, the fraction time may become shorter over time. It is also important to note that optimal bladder filling times may differ between patients, although standard procedures have been established and applied. Therefore, it is crucial to establish an appointment system by detecting and considering all these differences in daily practice, especially in centers that treat high numbers of patients per day.

For the 3%/3 mm criterion, the median gamma pass rate was 99.4% (range, 94.6–100%) in the present study. This result was consistent with the results of some of the previous studies that used the same criteria. Mönnich et al. and Yang et al. reported gamma pass rates of 99% (median) and 99.6% (mean), respectively (13, 14). Differently, de Leon et al. reported a partially lower gamma pass rate of 97.5% (mean) with the same criteria (12). The authors concluded that this difference may be related to the case mix and variations in practice.

In our rough assessment, the machine-associated treatment interruption rate was 2.9% for prostate cancer radiotherapy with 1.5 T MRI-LINAC. However, this rate decreased to 0.8% after the configuration upgrade. The existing literature on this topic is limited, with unclear information. Alongi et al. reported that no treatment interruptions occurred in their observational study, which included prostate cancer patients treated with 1.5 T MRI-guided SBRT (10). Considering the effects of this upgrade and possible future upgrades, it could be said that the machine-associated interruption rates might approach zero over time. Moreover, all the patients tolerated prostate cancer radiotherapy with 1.5 T MRI-LINAC well, without any grade III or higher acute genitourinary or gastrointestinal toxicity. Grade II toxicity (rectal pain and bleeding) was observed in only one patient. This patient underwent bilateral hip replacement, and his radiation treatment field angles had some limitations due to the presence of the prostheses that cause artifacts on CT images. It was difficult to determine whether this situation might have contributed to the development of grade II gastrointestinal toxicity in this patient. Importantly, it should be noted that MRI-guided radiotherapy looks like a reasonable solution for the application of prostate cancer radiotherapy in patients with bilateral hip replacements with a MR only workflow (15).

There are some limitations to this study. First, this is a retrospective study. Second, our patient series is heterogeneous and includes a small number of patients, because MRI-guided radiotherapy is a novel treatment option. Therefore, it is not possible to make final conclusions. On the other hand, this study represents an analysis of MRI-guided prostate radiotherapy that includes the largest number of fractions in the literature. And, this is the strength of our study compared with its previous counterparts in the literature.

## Conclusion

When the superior abdominal imaging capability, online adaptation ability, tolerable daily fraction time, excellent dose delivery success, additional future software/hardware upgrades, adequate patient tolerance, and easily manageable acute side effect rates are evaluated together, it is clear that MRI-guided prostate cancer radiotherapy is safe and feasible.

When the conditions of the radiotherapy center are appropriate, the 1.5 MRI-LINAC device can not only be used for ultra-hypofractionated regimens, but also as a conventional LINAC device during prostate cancer radiotherapy, even in the pelvic lymph node area. To better evaluate this promising new technology, high-quality studies with a larger number of patients and long-term results are warranted.

## Data availability statement

The raw data supporting the conclusions of this article will be made available by the authors, without undue reservation.

## Ethics statement

The studies involving human participants were reviewed and approved by Human Research Ethics committee of the Istinye University. The patients/participants provided their written informed consent to participate in this study. Written informed consent was obtained from the individual(s) for the publication of any potentially identifiable images or data included in this article.

## Author contributions

GT was the lead author. GT and MF designed and coordinated the study. NB, HS, YK, SA, BT, and MO participated in data collection. GT and MF participated in data analysis, article drafting, table/figure creation, and article revision. All authors contributed to the article and approved the submitted version.

## Acknowledgments

We would like to thank Editage (www.editage.com) for English language editing.

## Conflict of interest

The authors declare that the research was conducted in the absence of any commercial or financial relationships that could be construed as a potential conflict of interest.

## Publisher’s note

All claims expressed in this article a re solely those of the authors and do not necessarily represent those of their affiliated organizations, or those of the publisher, the editors and the reviewers. Any product that may be evaluated in this article, or claim that may be made by its manufacturer, is not guaranteed or endorsed by the publisher.

## References

[1] Siegel RL Miller KD Fuchs HE Jemal A. Cancer statistics, 2021. CA Cancer J Clin (2021) 71(1):7–33. doi: 10.3322/caac.21654 33433946

[2] Kontaxis C Bol GH Kerkmeijer LGW Lagendijk JJW Raaymakers BW. Fast online replanning for interfraction rotation correction in prostate radiotherapy. Med Phys (2017) 44(10):5034–42. doi: 10.1002/mp.12467 28703497

[3] Murray J Tree AC. Prostate cancer - advantages and disadvantages of Mr-guided rt. Clin Transl Radiat Oncol (2019) 18:68–73. doi: 10.1016/j.ctro.2019.03.006 31341979 PMC6630102

[4] Pathmanathan AU van As NJ Kerkmeijer LGW Christodouleas J Lawton CAF Vesprini D. Magnetic resonance imaging-guided adaptive radiation therapy: A "Game changer" for prostate treatment? Int J Radiat Oncol Biol Phys (2018) 100(2):361–73. doi: 10.1016/j.ijrobp.2017.10.020 29353654

[5] Mahmood F Johannesen HH Geertsen P Hansen RH. Repeated diffusion mri reveals earliest time point for stratification of radiotherapy response in brain metastases. Phys Med Biol (2017) 62(8):2990–3002. doi: 10.1088/1361-6560/aa5249 28306548

[6] Marks LB Yorke ED Jackson A Ten Haken RK Constine LS Eisbruch A. Use of normal tissue complication probability models in the clinic. Int J Radiat Oncol Biol Phys (2010) 76(3 Suppl):S10–9. doi: 10.1016/j.ijrobp.2009.07.1754 PMC4041542 20171502

[7] Brand DH Tree AC Ostler P van der Voet H Loblaw A Chu W. Intensity-modulated fractionated radiotherapy versus stereotactic body radiotherapy for prostate cancer (Pace-b): Acute toxicity findings from an international, randomised, open-label, phase 3, non-inferiority trial. Lancet Oncol (2019) 20(11):1531–43. doi: 10.1016/S1470-2045(19)30569-8 PMC6838670 31540791

[8] Winkel D Bol GH Kroon PS van Asselen B Hackett SS Werensteijn-Honingh AM. Adaptive radiotherapy: The elekta unity Mr-linac concept. Clin Transl Radiat Oncol (2019) 18:54–9. doi: 10.1016/j.ctro.2019.04.001 PMC6630157 31341976

[9] de Muinck Keizer DM Kerkmeijer LGW Willigenburg T van Lier A Hartogh MDD van der Voort van Zyp JRN. Prostate intrafraction motion during the preparation and delivery of Mr-guided radiotherapy sessions on a 1.5t Mr-linac. Radiother Oncol (2020) 151:88–94. doi: 10.1016/j.radonc.2020.06.044 32622779

[10] Alongi F Rigo M Figlia V Cuccia F Giaj-Levra N Nicosia L. 1.5 T Mr-guided and daily adapted sbrt for prostate cancer: Feasibility, preliminary clinical tolerability, quality of life and patient-reported outcomes during treatment. Radiat Oncol (2020) 15(1):69. doi: 10.1186/s13014-020-01510-w 32248826 PMC7092497

[11] Bertelsen AS Schytte T Moller PK Mahmood F Riis HL Gottlieb KL. First clinical experiences with a high field 1.5 T Mr linac. Acta Oncol (2019) 58(10):1352–7. doi: 10.1080/0284186X.2019.1627417 31241387

[12] de Leon J Crawford D Moutrie Z Alvares S Hogan L Pagulayan C. Early experience with Mr-guided adaptive radiotherapy using a 1.5 T Mr-linac: First 6 months of operation using adapt to shape workflow. J Med Imaging Radiat Oncol (2022) 66(1):138–45. doi: 10.1111/1754-9485.13336 34643065

[13] Monnich D Winter J Nachbar M Kunzel L Boeke S Gani C. Quality assurance of imrt treatment plans for a 1.5 T Mr-linac using a 2d ionization chamber array and a static solid phantom. Phys Med Biol (2020) 65(16):16NT01. doi: 10.1088/1361-6560/aba5ec 32663819

[14] Yang B Wong YS Lam WW Geng H Huang CY Tang KK. Initial clinical experience of patient-specific qa of treatment delivery in online adaptive radiotherapy using a 1.5 T Mr-linac. BioMed Phys Eng Express (2021) 7(3). doi: 10.1088/2057-1976/abfa80 33882471

[15] Felisi M Monti AF Lizio D Nici S Pellegrini RG Riga S. Mri only in a patient with prostate cancer with bilateral metal hip prostheses: Case study. Tumori (2021) 107(6):NP41–NP4. doi: 10.1177/0300891621997549 33629653

